# Beckwith‐Wiedemann syndrome with macroglossia as the most significant manifestation: A case report

**DOI:** 10.1002/ccr3.4479

**Published:** 2021-07-06

**Authors:** Shatha Lamfoon, Sondos Abuzinada, Ahmad Yamani, Nada Binmadi

**Affiliations:** ^1^ Oral Diagnostic Sciences Department Faculty of Dentistry King Abdulaziz University Jeddah Saudi Arabia; ^2^ Oral and Maxillofacial Surgery Department Faculty of Dentistry King Abdulaziz University Jeddah Saudi Arabia

**Keywords:** Beckwith‐Wiedemann syndrome, glossectomy, macroglossia

## Abstract

Beckwith‐Wiedemann syndrome is a complex multisystem disorder that requires collaboration of medical and dental teamfor its diagnosis and management. We present a dental overview and an update of the clinical and molecular diagnoses of Beckwith‐Wiedemann syndrome and its management with emphasis on macroglossia.

## Graphical abstract

A 1‐year‐old male infant diagnosed with a rare congenital disease Beckwith‐Wiedemann syndrome (BWS) since birth presented with macroglossia, hemihypertrophy, and mild bilateral nephromegaly. Surgical debulking of the enlarged tongue was performed. This report emphasized the significance of tongue enlargement in the clinical diagnosis of BWS and case management.

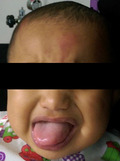

## 1 INTRODUCTION

Beckwith‐Wiedemann syndrome (BWS), originally termed exomphalos, macroglossia, and gigantism syndrome, was independently described by Beckwith, an American pathologist, and Wiedemann, a German pediatrician, in 1960.^1^ BWS is an overgrowth syndrome that results in the overgrowth of any part of the body as one of its three main presenting features during infancy, along with abdominal wall defects and macroglossia.^2^ Patients may also present with other features of varying severities such as ear abnormalities, hemihyperplasia, enlarged abdominal organs, neonatal hypoglycemia, and increased predisposition to embryonal tumors during early childhood.^2^, ^3^, ^4^

Variability in the mode of inheritance and clinical presentation of BWS has led to an underestimation of the exact prevalence and severity of the disease.^3^ Nonetheless, it is considered the most common congenital overgrowth disorder, despite its relatively low prevalence of 1 in 10,340 live births as reported in different ethnicities.^5^ Approximately 5% of BWS cases are sporadic, while 40% of the cases are inherited.^6^ The complex underlying genetic mechanisms of BWS involving molecular aberrations of the genes within chromosome 11p15.5 include translocation, duplication, or inversion of this chromosome.^3^ The most extensively studied genes of the chromosome 11p15.5 region implicated in BWS are *potassium voltage‐gated channel subfamily Q member 1 (KCNQ1OT1), insulin‐like growth factor 2 (IGF2), and cyclin‐dependent kinase inhibitor 1C* *(CDKN1C)* and imprinted maternally expressed transcript (*H19)* genes.^5^, ^6^ Epigenetic changes were also seen on chromosome 11p15.5 as a defect in the methylation process at the maternal and paternal alleles, which is a major characteristic of imprinted genes associated with BWS.^7^

To address the phenotypic variety of BWS, an international consensus statement in the study by Brioude et al^6^ recommended the use of a clinical scoring system for the diagnosis. The scoring system relies on the cardinal and suggestive features. Cardinal features are those that when present are strongly suggestive of BWS; thus, two points are assigned for each feature. These include macroglossia, omphalocele, lateralized overgrowth, hyperinsulinism, bilateral Wilms tumors, and specific pathological findings such as placental mesenchymal dysplasia or adrenal cytomegaly.^6^ By contrast, suggestive features are those characterized as being independent of the general pediatric population, such as birth weight greater than two standard deviations above the mean, ear creases or pits, polyhydramnios or placentomegaly, facial nevus simplex, transient hypoglycemia, nephromegaly or hepatomegaly, embryonal tumors, and umbilical hernia or diastasis recti.^6^ Each of these features was assigned one point. Based on this scoring scheme, a patient with a score of ≥4 satisfied the clinical diagnosis of classical BWS.^6^ A multidisciplinary team is often recommended for the management and care for a patient with BWS depending on both their phenotypic presentation and molecular subtype.^6^

The complex manifestations of the disease require different management protocols to provide coordinated health care for patients. One of the target features to be addressed is macroglossia, since approximately 90% of patients diagnosed with BWS exhibit this feature.^6^ Similarly, most cases of macroglossia during childhood are due to BWS.^6^ Approximately 40% of children diagnosed with BWS undergo a surgical tongue reduction because it may otherwise lead to functional difficulties with feeding, breathing, drooling, and speech as well as affect facial appearance.^5^ The goal of the surgical procedure is to reduce tongue bulk while preserving its normal shape and improving function.^8^

In this paper, a case of BWS is presented to create further awareness and highlight the clinical features of BWS mainly macroglossia and diagnosis guidelines. In addition, we highlighted the management of macroglossia, which is the most significant complications associated with BWS.

## 2 CASE DESCRIPTION

**FIGURE 1:**
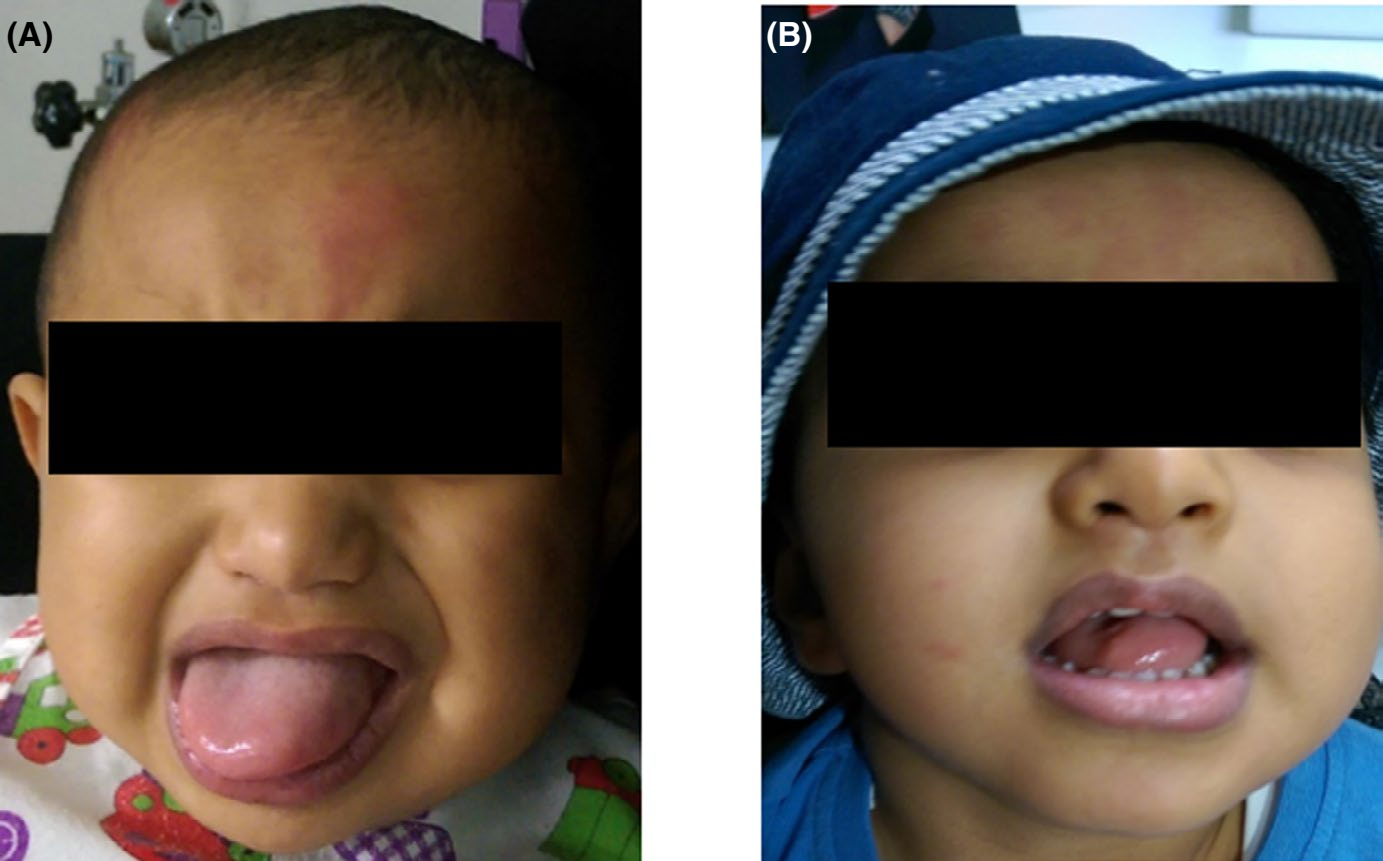
Patient diagnosed with Beckwith‐Wiedemann syndrome. (A) Clinical presentation of macroglossia at age 1 year; and (B) Patient at 1‐year follow‐up after the surgery (2‐  years‐old), showing improvement.

A 1‐year‐old male infant who had been diagnosed after birth by his pediatrician with BWS, with macroglossia as the first feature that led to the diagnosis, was referred to the Oral and Maxillofacial Department at the faculty of dentistry of King Abdul Aziz University. On clinical examination that was performed by the maxillofacial surgeon, the patient presented with macroglossia, which led to an inability to close his mouth, interference with occlusion, feeding and swallowing difficulties, and drooling (Figure 1A), and hemihypertrophy of the left side of the body. The patient has no family history of similar condition. He underwent full assessment by a multidisciplinary team comprising pediatrician, genetic specialists, oral and maxillofacial surgeons, pathologists, pedodontists, and orthodontists to assess his status and treatment options.

To confirm the diagnosis clinically, ultrasonography showed mild hepatomegaly and mild bilateral nephromegaly with no other detectable abnormalities. Accordingly, liver and kidney functions were assessed including the alpha fetoprotein level for tumor screening, and all values were within the normal limit. His vital signs and blood sugar level were normal. No molecular confirmation was performed for this case so far. These findings support the clinical diagnosis of BWS according to the scoring system, including two cardinal features and one suggestive feature. Our patient's score was 5, ie, 2 points for macroglossia, 2 points for lateralized overgrowth, and 1 point for hepatomegaly and nephromegaly.

**FIGURE 2:**
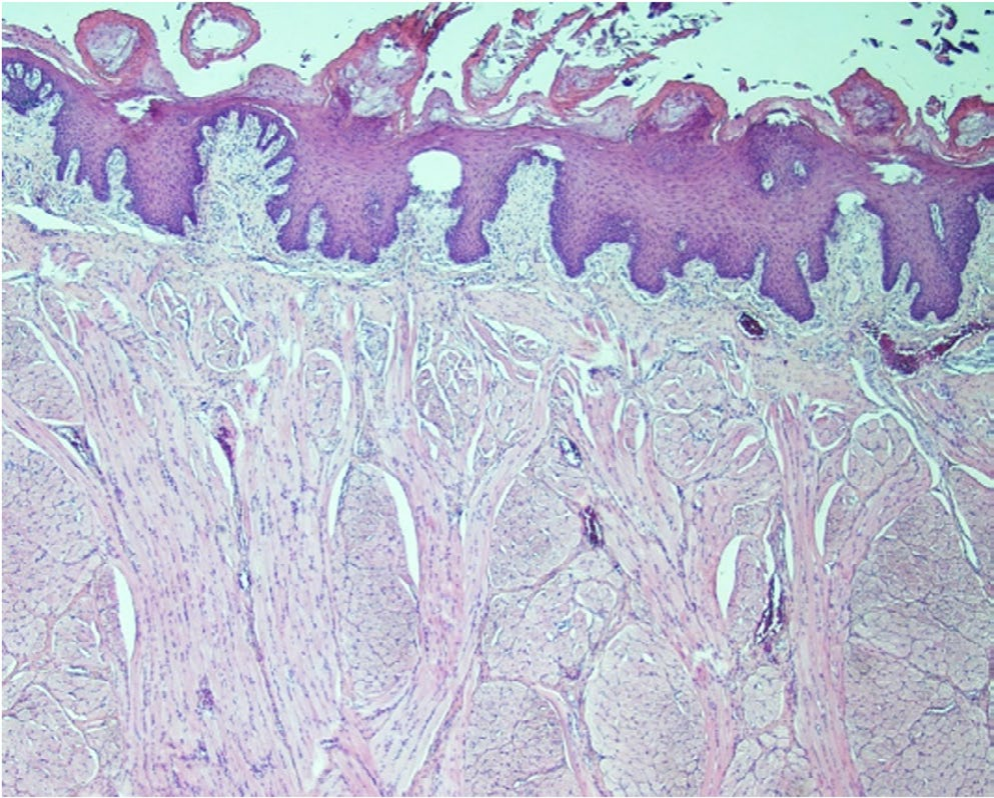
Histopathological image of excised tongue tissue from patient showing muscular hyperplasia

Surgical debulking of the enlarged tongue was performed by oral and maxillofacial surgeons and was performed at age 1 year under general anesthesia because he had breathing and swallowing difficulties. The patient was placed in the supine position with nasal intubation, and a modified reduction glossectomy was performed using the stellate anterior wedge procedure. A specimen of the tongue was submitted to the Oral and Maxillofacial Pathology Department. Histopathology using hematoxylin and eosin staining revealed muscular hyperplasia (Figure 2).

At discharge and after 7 days of follow‐up, the patient was well. During the follow‐up at age 2 years (1 year after the surgery), difficulties in airway and speech were not observed (Figure 1B). In concordance, the patient undergoes periodic follow‐up with his pediatrician to assess enlargement of visceral organs and liver and kidney functions.

## 3 DISCUSSION

BWS is considered the most common congenital overgrowth disorder. Patients with BWS have diverse clinical manifestations. With this dilemma, a scoring system can aid in the diagnosis of patients as in our case. A clinical score of ≥4 based on the diagnostic scheme, with at least two cardinal features, is sufficient to satisfy the clinical diagnosis of classical BWS without molecular confirmation, as in our patient.^6^ A patient with atypical BWS may meet the clinical criteria for having a clinical diagnosis of BWS if the score was ≥4, but it depends more on the suggestive features.^6^ According to an international consensus statement, genetic testing and molecular confirmation are recommended for patient with a score of ≥2 including classical BWS with a score of ≥4.^6^ As some of the features could be present with other alternative diagnosis and the tumor risk in BWS depend on the nature of molecular defect found.^6^, ^9^

Macroglossia is one of the primary manifestations of BWS diagnosis, reported in nearly 90% of BWS cases.^6^ It is diagnosed based on morphology and on whether growth, feeding, functional, or psychological problems arise from tongue protrusion.^4^, ^10^ Among all other reported cases, only one case was reported to have oral tongue polyp instead of diffuse enlargement.^11^ Apparent skeletal muscle hyperplasia (Figure 2) resulted in macroglossia in our case. Recent studies have proved that macroglossia in BWS histologically showed a true muscular hyperplasia rather than hypertrophy of the muscle due to an increase in the number of skeletal muscle fibers without an increase in the diameter of the fiber itself compared with the normal. Moreover, the expression of IGF‐2 is high in hyperplastic muscles, and there are epigenetic changes in the hypermethylation of the paternal uniparental disomy.^2^, ^12^ Tongue reduction is the optimal choice of treatment of BWS patient with macroglossia to reduce drooling and feeding, breathing, and speech difficulties and prevent problems associated with facial appearance and occlusion.^12^ The appropriate timing for surgery is before age 2 years to obtain favorable functional and esthetic results.^5^, ^13^ In the present case, we performed the tongue reduction procedure at age 1 year to reduce central tongue bulk and tongue length and restore the anterior contour.^8^ The patient should be followed up by a dental team for recurrence, facial appearance, and ensure proper teeth eruption and occlusion.^2^ Further observational studies should be conducted to emphasize complications after surgery and how they could be avoided.

Aside from the functional difficulties caused by macroglossia, BWS highly predisposes affected children to cancerous and non‐cancerous tumors. Hepatoblastoma, a form of liver cancer, and Wilms tumor, a kidney cancer, can develop. The risk of severity of these tumors depends on clinical and molecular findings.^5^ Therefore, follow‐up is recommended to strictly screen for tumors or other manifestations by abdominal ultrasonography as first‐line investigation for Wilms tumor and hepatoblastoma and to measure serum alpha fetoprotein levels every 3 months until age 8 and 4 years, respectively.^14^

In the present case, the patient obtained a score of 5, thus confirming the diagnosis of BWS. The tongue reduction procedure is the best treatment option that proved effective in reducing complications. However, some patients may require a second reduction glossectomy at an older age. Follow‐up and monitoring by a dental team is important to maintain function and restore the aesthetic appearance of the patient.

## CONFLICTS OF INTERESTS

None declared.

## AUTHOR CONTRIBUTION

All authors have made substantial contributions: SL: collected and interpreted data and wrote the manuscript. SA: obtained patient consent, collected data, treated the patient, and revised the manuscript. AY: revised the article and approved the final version for submission. NB: involved in the analysis and interpretation of data and critically revised the manuscript.

## ETHICAL APPROVAL

Informed consent was obtained from the parent regarding the publication of images and data.

## Data Availability

Data are available on request from the authors.

## References

[1] Chen H . Atlas of genetic diagnosis and counseling. 3rd ed. New York, NY: Springer‐Verlag New York; 2017:109‐113.

[2] Yamada T , Sugiyama G , Higashimoto K , et al. Beckwith‐Wiedemann syndrome with asymmetric mosaic of paternal disomy causing hemihyperplasia. Oral Surg Oral Med Oral Pathol Oral Radiol. 2019;127:e84‐e88. 30340909 10.1016/j.oooo.2018.07.053

[3] Galerneau F . 109 ‐ Beckwith‐Wiedemann syndrome. In: Copel JA , D’Alton ME , Feltovich H , et al, eds. Obstetric imaging: fetal diagnosis and care (second edition). Amsterdam, The Netherlands: Elsevier; 2018:462‐466.e1.

[4] Matamala GN , De Los Ángeles Fernández Toro M , Ugarte EV , Mendoza ML . Beckwith‐Wiedemann syndrome: presentation of a case report. Med Oral Patol Oral Cir Bucal. 2008;13:640‐643. 18830172

[5] Wang KH , Kupa J , Duffy KA , Kalish JM . Diagnosis and management of Beckwith‐Wiedemann syndrome. Front Pediatr. 2019;7:562. 32039119 10.3389/fped.2019.00562 PMC6990127

[6] Brioude F , Kalish JM , Mussa A , et al. Expert consensus document: clinical and molecular diagnosis, screening and management of Beckwith‐Wiedemann syndrome: an international consensus statement. Nat Publ Gr. 2018;14:229‐249. 10.1038/nrendo.2017.166 PMC6022848 29377879

[7] Maher ER , Reik W , Tycko B . Beckwith‐Wiedemann syndrome: imprinting in clusters revisited. J Clin Invest. 2000;105:247‐252. 10675349 10.1172/JCI9340 PMC517490

[8] Heggie AAC , Vujcich NJ , Portnof JE , Morgan AT . Tongue reduction for macroglossia in Beckwith Wiedemann syndrome: review and application of new technique. Int J Oral Maxillofac Surg. 2013;42:185‐191. 23041202 10.1016/j.ijom.2012.09.003

[9] Maas SM , Vansenne F , Kadouch DJM , et al. Phenotype, cancer risk, and surveillance in Beckwith‐Wiedemann syndrome depending on molecular genetic subgroups. Am J Med Genet A. 2016;7:2248‐2261. 10.1002/ajmg.a.37801 27419809

[10] Matsune K , Miyoshi K , Kosaki R , Ohashi H , Maeda T . Taste after reduction of the tongue in Beckwith‐Wiedemann syndrome. Br J Oral Maxillofac Surg. 2006;44:49‐51. 15896892 10.1016/j.bjoms.2005.03.015

[11] Kujan O , Raheel SA , King D , Iqbal F . Oral polyp as the presenting feature of Beckwith‐Wiedemann syndrome in a child. BMJ Case Rep. 2015;2015:bcr2015210758. 10.1136/bcr-2015-210758 PMC4693137 26323977

[12] Oyama Y , Nishida H , Kobayashi O , Kawano K , Ihara K , Daa T . Macroglossia in Beckwith‐Wiedemann syndrome is attributed to skeletal muscle hyperplasia. Case Rep Dent. 2020;2020:8871961. 33204545 10.1155/2020/8871961 PMC7652604

[13] Mussa A , Russo S , De Crescenzo A , et al. Prevalence of Beckwith‐Wiedemann syndrome in North West of Italy. Am J Med Genet Part A. 2013;161:2481‐2486. 10.1002/ajmg.a.36080 23918458

[14] Tan TY , Amor DJ . Tumour surveillance in Beckwith‐Wiedemann syndrome and hemihyperplasia: a critical review of the evidence and suggested guidelines for local practice. J Paediatr Child Health. 2006;42:486‐490. 16925531 10.1111/j.1440-1754.2006.00908.x

